# Odor Identification by Parkinson's Disease Patients Tested by Using Sniffin' Sticks versus Natural Spices

**DOI:** 10.1155/2022/2272691

**Published:** 2022-04-28

**Authors:** Florence Baert, Geertrui Vlaemynck, Jarissa Maselyne, Christophe Matthys

**Affiliations:** ^1^Department Technology and Food, Flanders Research Institute for Agriculture, Fisheries and Food, Melle, Belgium; ^2^Clinical and Experimental Endocrinology, Department of Chronic Diseases and Metabolism, KU Leuven, O&N I, Leuven, Belgium; ^3^Department of Endocrinology, University Hospitals Leuven, Campus Gasthuisberg, Leuven, Belgium

## Abstract

**Introduction:**

Hyposmia is a frequent symptom of Parkinson's disease (PD), which greatly impacts patients' flavor perception and their quality of life. However, PD patients recognize some odors better than others. Identifying which food odors are still recognized by PD patients may be useful for flavor enhancement. Our aim was to evaluate the olfactory identification of Sniffin' Sticks and spice odorants in PD patients and healthy controls (HC), to identify the impact of synthetic odorants compared with real-life food and the impact of odor familiarity and pleasantness on odorant identification in PD patients.

**Methods:**

Sniffin' Sticks odorant identification was evaluated in 80 PD patients and 105 age-matched HC. In a subset, the spice odorant identification was evaluated.

**Results:**

The mean total score was higher for the Sniffin' Sticks than for the spice odor identification test in all participants (55.4% versus 22.5%). Sniffin' Sticks orange, peppermint, rose, and fish odorants were best correctly identified by PD patients, by 62.5, 53.8, 52.9, and 57.5%, respectively. Of the spice odor identification test, garlic and “no stimulus” were best correctly identified by PD patients, by, respectively, 38.2 and 67.6%. HC identified most Sniffin' Sticks odorants and spices better than PD patients. Odorant familiarity determined real-life food odorant identification.

**Conclusion:**

This study demonstrates that some food odorants, both the commercial Sniffin' Sticks as natural odorants, are still recognized by PD patients. Sniffin' Sticks were better recognized compared with real-life odorants, by both HC and PD patients. Odorant familiarity determined PD patients' odorant identification; therefore, familiar food odorants may have potential for a future flavor enhancement. *Implications*. This is the first study, to our knowledge, to evaluate real-life food odor identification in PD patients. Our results provide a first step towards patient-appropriate flavor enhancement strategies in PD.

## 1. Introduction

Though recognized as a movement disorder, Parkinson's disease (PD) is characterized by both motor and non-motor symptoms. Some non-motor symptoms such as hyposmia, with a prevalence of 70–90%, precede the onset of motor symptoms by many years [1]. Currently, there is no treatment for olfactory impairment in PD. The involvement of altered neurotransmitter levels and neuropathological changes has been proposed as underlying mechanisms [2]. The loss of sense of smell greatly impacts the patient's quality of life (QoL), as a result of insecurities regarding personal hygiene, lack of hazard awareness (e.g., burning smell and gas leaks), and reduced food enjoyment [3, 4]. Olfactory stimuli are important food cues that can increase appetite and play an important role in flavor perception [5, 6]. It has been demonstrated that exposure to olfactory stimuli results in increased food intake [7]. Consequently, in PD the olfactory impairment may influence food palatability, food enjoyment, and even food intake.

Although previous studies [8–10] showed odor identification deficits in PD patients compared with age-matched healthy controls (HC), various studies also demonstrated that some odorants are still recognized by PD patients [11–13]. This phenomenon is described as selective hyposmia; however, the odorants that differentiate PD patients from healthy volunteers differ between studies [14, 15]. This may be influenced by environmental, cultural, or genetic factors [14, 16]. Though the selective hyposmia may not be pathology-specific, it still demonstrates that, despite olfactory impairment, PD patients are able to detect certain scents. Furthermore, studies have demonstrated that olfactory training can improve odor discrimination and identification in hyposmia [9, 17].

The use of synthetic everyday odorants, as in the Sniffin' Sticks [18], may have a different impact on odor recognition compared with the use of natural, complex, so-called *real-life*, food odorants (e.g., herbs and spices as used in food preparation). The identification of odors may be useful in the development of aroma or flavor boosters for PD patients. The use of aroma or flavor boosters might improve food palatability and thereby increase both food enjoyment and food intake in PD.

The aim of this study was to evaluate the olfactory identification of Sniffin' Sticks odors and *real-life* spice odors in PD patients and HC. Furthermore, we hypothesized that both familiarity and pleasantness of the *real-life* spice odorant would be associated with its identification by PD patients and HC.

## 2. Materials and Methods

### 2.1. Study Design and Population

An observational, cross-sectional study was conducted between February 2017 and December 2019 in PD patients and HC of similar age. The study consisted of 2 parts to evaluate the olfactory identification of Sniffin' Sticks and *real-life* food odors, i.e., spices of the spice odor identification test [19, 20]. In the first part of the study, 185 participants (105 HC and 80 PD patients) completed the Sniffin' Sticks odor identification test and a general questionnaire regarding health and medication intake. In the second part of the study, a conveniently chosen subset of the original sample of participants (*n* = 87 (34 PD patients and 53 HC), ∼47%, Table 1) carried out a spice odor identification test [19, 20] and a Mini- Nutritional Assessment-Short Form (MNA-SF) [21, 22] on top of the Sniffin' Sticks test. In accordance with the advice of the Ethics Committee of the University of Leuven, participant data were anonymized, and therefore, no written informed consent was obtained. However, all participants provided oral informed consent prior to participating in the study. The study protocol complied with the Helsinki declaration and was approved by the Ethics Committee of the University of Leuven (S61771-B322201837590).

PD patients and HC (partners or caregivers of PD patients) were recruited in collaboration with the patient's organization “Parki's Cook Atelier” and regional departments of the patient's organization “Flemish Parkinson Association.” Inclusion criteria for PD patients were as follows: age above 55 years and self-reported diagnosis of PD, cross-checked by the research team. The inclusion criterion for HC was an age above 55 years. Before the start of the test, the test procedure was clearly explained to all participants. Exclusion criteria for both PD patients and HC were as follows: atypical or secondary Parkinson, acute rhinosinusitis, dementia, or any other cognitive or psychiatric disorders inhibiting study participation. If participants were unable to understand or perform this test independently, they were excluded from the sample.

### 2.2. Odor Identification Tests

**Figure 1:**
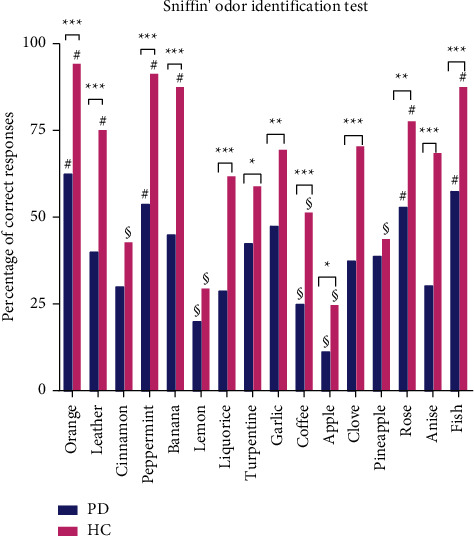
Responses of Parkinson's patients and healthy controls for the Sniffin' Sticks odor identification test; HC, healthy controls; PD, Parkinson's disease patients; ^*∗*^*p* < 0.05; ^*∗∗*^*p* < 0.01; ^*∗∗∗*^*p* < 0.001; # an odorant that is better recognized compared with the other odorants within the specific population group; §, an odorant that is least recognized compared with the other odorants within the specific population group.

Participants were asked to refrain from smoking, eating, or drinking for 1 hour prior to the test, and only water was allowed. To analyze the participant's olfactory identification ability, using synthetic stimuli, the 16-item Sniffin' Sticks (identification test 16, blue) was used [18]. This test consists of 16 fragrance pens, with everyday scents (see Figure 1) such as the smell of leather or pineapple. The participant was offered the fragrance pens one by one and had to identify the smell by means of multiple forced choice (4 options for each odorant).

**Figure 2:**
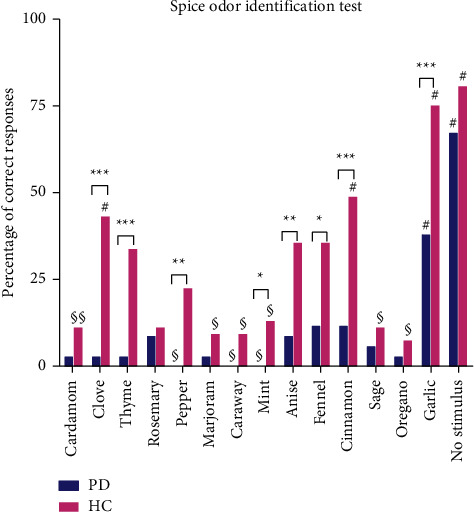
Responses of Parkinson's patients and healthy controls for the spice odor identification test; HC, healthy controls; PD, Parkinson's disease patients; ^*∗*^*p* < 0.05; ^*∗∗*^*p* < 0.01; ^*∗∗∗*^*p* < 0.001; # an odorant that is better recognized compared with the other odorants within the specific population group; §, an odorant that is least recognized compared with the other odorants within the specific population group.

In the conveniently chosen subset of the original sample, the spice odor identification test with dried spices as odorants was used, following a previously published protocol [19]. Prior to the test, the participant was asked to estimate his own sense of smell (5-point scale from very good to very bad, coded from 0 to 4). The spice odor identification test contains 15 olfactory stimuli (14 dried spices and 1 blank; see Figure 2). The stimuli consisted of 40 ml containers filled with a teaspoon of dried spices. The containers were closed and wrapped in aluminum foil to hide their contents. Participants were offered the samples one by one, without the lid, and were asked to smell. Next, the participant had to identify the odor from an alphabetical list of 17 response options (14 stimuli used, 2 distraction stimuli, and the “no odor” option). The same answer options were used for each sample. A visual aid of the 17 response options was given to the participants. Per scent, the participant had to report the perceived pleasantness (9-point scale from very pleasant to very unpleasant, coded from 0 to 8) and familiarity (5-point scale from very familiar to unfamiliar, coded from 0 to 4).

Both tests follow a “forced choice procedure,” in which the participant is obliged to choose an answer. In this subset of participants, nutritional screening using the MNA-SF was carried out.

### 2.3. Data Analysis

Collected data from the Sniffin' Sticks were analyzed according to the kit's guidelines, and each correctly identified odor obtained a score of 1, with a total score as the sum of all correctly identified odors ranging from 0 to 16. A total score lower than or equal to 8 was defined as anosmia, and a score between 8 and 11 was defined as hyposmia, whereas a score above 11 was defined as normosmia [18]. In the spice odor identification test, each correctly identified odor obtained a score of 1, with a total score as the sum of all correctly identified odors ranging from 0 to 15. Because of the similarity in scent of anise and fennel seeds, when participants confused the 2 scents with each other, answers were considered correct. The collected data of MNA-SF were analyzed according to the guidelines. A screening score equal to or below 7 was defined as malnourished, a score of 8 to 11 was defined as at risk for malnutrition, and a score of 12 to 14 was defined as a normal nutritional status [22]. The Hoehn and Yahr scale was collected in 22 PD patients, by contacting their neurologist. The Hoehn and Yahr scale allows the assessment of motor symptoms in PD. Figures were created using GraphPad Prism 9.

### 2.4. Statistical Analysis

Statistical analyses were carried out according to previously published studies [19, 20]. The normality of data was assessed using histograms and the Shapiro–Wilk test. Possible differences in sex, smoking, self-estimated sense of smell, and medication intake between PD and HC were analyzed using Pearson's chi-square test and Fisher's exact test (when counts were below 5). Possible differences in age, BMI, and MNA-SF score between PD and HC were analyzed using Student's *t*-test or the Mann–Whitney *U* test, depending on normality. Possible correlations between olfactory identification test scores and continuous covariates were assessed using Pearson's or Spearman's correlations. Possible correlations between olfactory identification test scores and categorical covariates were analyzed using the Mann–Whitney *U* test and Kruskal–Wallis test. Potential correlations between mean pleasantness, mean familiarity, and the percentage of correct identification of an odorant were analyzed using the Spearman rank analysis. Hereby, the “no stimulus” sample was not taken into consideration. To analyze whether there was a difference in familiarity/pleasantness between correct and wrong answers of odor identification, regardless of the odorant, a Kruskal–Wallis test on the raw data was done. Hereby, the “no stimulus” sample was not taken into consideration. Post hoc analysis was done through pairwise comparisons with the Bonferroni correction. To analyze which odorants were better recognized within PD patients or HC, Pearson's chi-square test was used. When the chi-square test was significant, overrepresentation and underrepresentation of recognized odorants were based upon standardized Pearson's residuals. To compare the test scores of the Sniffin' test and the spice odor identification test within the subset of participants, a Wilcoxon signed-rank test was used. Statistical significance was determined as *p* < 0.05. Analyses were carried out using RStudio 1.1.456.

## 3. Results

According to the Sniffin' Sticks test score, 73.8% of PD patients had anosmia, 23.8% had hyposmia, and 2.5% had normosmia (Table 1). In contrast, 21.9% of HC had anosmia, 43.8% had hyposmia, and 34.3% of HC had normosmia (Table 1).

No difference in the identification of cinnamon, lemon, and pineapple was observed between PD patients and HC in the Sniffin' Sticks test (Figure 1). Other scents of the Sniffin' Sticks test were significantly better identified by HC than PD patients. In PD patients, orange, peppermint, rose, and fish odorants were best recognized out of all odorants (Figure 1), whereas apple, lemon, and coffee were least recognized. In HC, orange, peppermint, fish, banana, rose, and leather odorants were best recognized, while apple, lemon, coffee, pineapple, and cinnamon were the least recognized odorants by HC (Figure 1).

The total score of the Sniffin' Sticks identification test was significantly associated with PD diagnosis (*U* = 7142, *p* < 0.001), sex (*U* = 5206, *p* = 0.008), and age (*r*_*s*_ = −0.18, *p* = 0.02) in all participants (Table 1). A trend (*U* = 2069, *p* = 0.06) towards an association between Sniffin' Sticks score and antidepressant intake in all participants was observed (Table 1). Within PD patients, a trend (*r*_*s*_ = -0.23, *p* = 0.06) towards an association between Sniffin' Sticks score and disease duration was observed (Table 1). Also, a trend (*U* = 354.5, *p* = 0.08) towards an association between PD medication intake and Sniffin' Sticks score in PD patients was observed. No association (H (6) = 4.51, *p* = 0.61) was found between the Hoehn and Yahr scale and the Sniffin' Sticks score (Table 1). Within HC, age (*r*_*s*_ = −0.33, *p* < 0.001), intake of anti-inflammatory drugs (*U* = 817, *p* = 0.03), and intake of medication for high cholesterol levels (*U* = 1292, *p* = 0.03) were associated with the Sniffin' Sticks score (Table 1).

**Figure 3:**
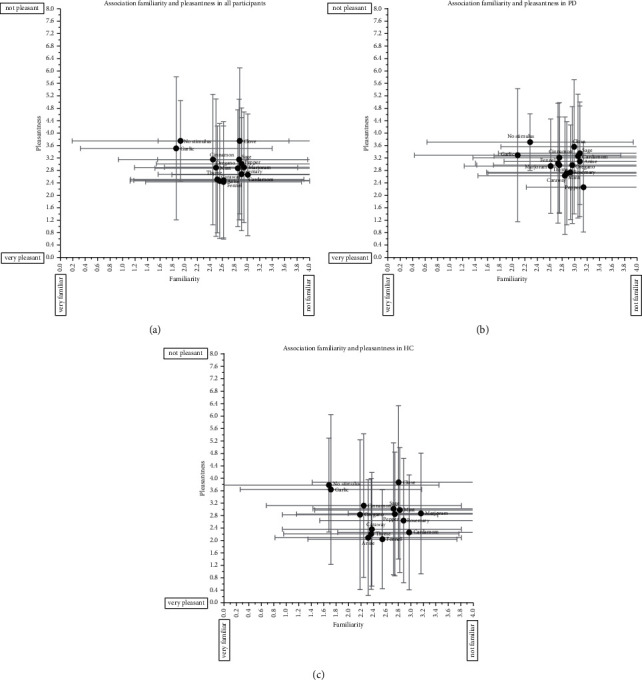
Correlation between mean pleasantness and mean familiarity of the spice odorants, in all participants (a), in Parkinson's disease patients (b), and healthy controls (c). Data are presented as mean ± SD; PD, Parkinson's disease, HC, healthy controls.

**Figure 4:**
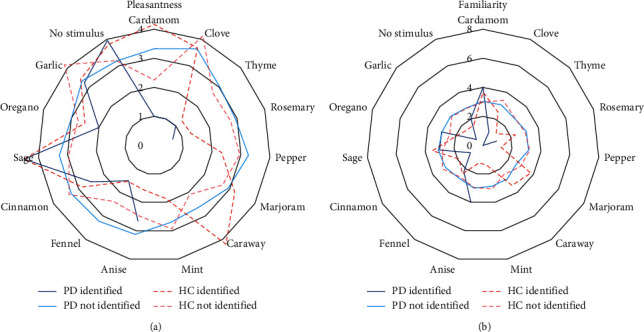
Correlation between pleasantness (a) and familiarity (b) of an odorant and its identification by Parkinson's patients and healthy controls; results are shown as mean; PDidentified, Parkinson's patients who correctly identified the odorant; PDnotidentified, Parkinson's patients who did not correctly identify the odorant; HCidentified, healthy controls who correctly identified the odorant; HCnotidentified, healthy controls who did not correctly identify the odorant; in Figure 3(a), 0 represents very pleasant and 4 represents not pleasant; in Figure 3(b), 0 represents very familiar, whereas 8 represents not familiar.

In the spice odor identification test, no difference in the identification of cardamom, rosemary, marjoram, caraway, sage, oregano, and “no stimulus” was found between PD patients and HC (Figure 2). Other odorants were all significantly better recognized by HC compared with PD. Garlic and “no stimulus” were best correctly identified in PD patients, while pepper, caraway, and mint were the least correctly identified odorants by PD patients, as they were not identified in this group (Figure 2). “No stimulus,” garlic, cinnamon, and clove were odorants best correctly identified by HC, whereas oregano, caraway, and marjoram were the three least correctly identified odorants by HC (Figure 2). The mean pleasantness of odorants was not correlated with their mean familiarity in all participants (*r*_*s*_ = −0.04, *p* = 0.90), within PD patients (*r*_*s*_ = 0.34, *p* = 0.23), or within HC (*r*_*s*_ = −0.02, *p* = 0.95); see also Figures 3(a)–3(c). An overall association (H (4) = 159.91, *p* < 0.001) was found between pleasantness and familiarity, regardless of the odorant. If one of the first two familiarity categories (“very familiar” and “familiar”) was chosen for an odorant, the odorant was regarded as more pleasant compared with the other 3 categories (“relatively familiar,” “a little familiar,” and “not familiar”). The mean pleasantness of an odorant was not correlated with its mean identification by all participants (*r*_*s*_ = 0.35, *p* = 0.22), PD patients (*r*_*s*_ = 0.35, *p* = 0.22), and HC (*r*_*s*_ = 0.26, *p* = 0.38); see Figure 4(a). However, an overall association (H (1) = 9.65, *p* = 0.002) was found between pleasantness and identification, regardless of the odorant. Participants who correctly identified odorants reported those odorants as more pleasant. The mean familiarity of an odorant was not correlated with its mean identification by all participants (*r*_*s*_ = −0.35, *p* = 0.22), PD patients (*r*_*s*_ = −0.36, *p* = 0.21), and HC (*r*_*s*_ = −0.39, *p* = 0.71); see Figure 4(b). However, an overall association regardless of odorant (H (1) = 118.75, *p* < 0.001) was found. Participants who correctly identified odorants reported those odorants as more familiar.

A significant association (H (4) = 21.01, *p* < 0.001) was found between self-estimated sense of smell and the test score in all participants of the spice odor identification test. The spice odor identification test score significantly (*U* = 1517, *p* < 0.001) differed between PD and HC. The test score was also associated with sex (*U* = 1331.5, *p* < 0.001) and age (*r*_*s*_ = −0.33, *p* = 0.002) in all participants. Within PD patients, a significant correlation (*r*_*s*_ = −0.37, *p* = 0.03) between age and test score was found. No association (H (6) = 9.87, *p* = 0.13) was found between the Hoehn and Yahr scale and test score. Within HC, the test score was associated with age (*r*_*s*_ = −0.34, *p* = 0.01). Within HC, trends towards a correlation between the spice odor identification test score and sex (*t* (31.9) = 2.03, *p* = 0.05), self-estimated sense of smell (H (3) = 7.71, *p* = 0.05), hypertension medication intake (*U* = 451, *p* = 0.06), and high cholesterol medication intake (*U* = 437, *p* = 0.07) were observed.

In the subset of participants who performed both tests, the mean total score of the Sniffin' Sticks test was significantly higher than the mean total score of the spice odor identification test in all participants (55.4% versus 22.5% (*Z* = −8.1, *p* < 0.001)), in PD patients (40.8% versus 8.2% (*Z* = −5.1, *p* < 0.001)), and in HC (64.7% versus 29.7% (*Z* = −6.3, *p* < 0.001)).

## 4. Discussion

Our results demonstrated that Sniffin' Sticks odors were more easily recognized compared with the *real-life* odorants of the spice odor identification test. It could, however, not be investigated whether this was a result of odorant concentration or other factors. Overall familiarity and pleasantness were associated with theidentification of *real-life* spice odorants by PD patients and HC;for the Sniffin' Sticks, this was not assessed.

As expected, PD patients had a reduced sense of smell compared with HC. Our results of median Sniffin' Sticks test score were similar to those of Zhao et al. in both PD patients (our results showed a median score of 6 versus a median score of 7) and HC (a median score of 11 versus a median score of 10) [23]. The results by Mahlknecht et al. indicated a mean Sniffin' Sticks test score for PD patients from 2 test centers similar to our results (6.8 ± 3.1 and 7.4 ± 3.0 versus 6.2 ± 3.1) [24]. Our results showed that 97.6% of PD patients had a reduced sense of smell (73.8% anosmic and 23.8% hyposmic), similar to the findings of Casjens et al., who reported an olfactory impairment in 93.3% of PD patients (56.8% anosmic and 36.5% hyposmic). The portion of HC that had a normal sense of smell was similar to Casjens et al. (34.3% versus 31.1%), whereas our results of anosmia in HC were higher compared with theirs (21.9% versus 6.8%) [25]. This difference might be due to an age difference; our maximum reported age was 84 in both PD and HC, whereas in Casjens et al. it was 73 and 72, respectively [25]. Medication intake might also explain these differences in the smelling capacity of participants; however, medication intake was not reported by Casjens et al. [25]. Yet, our results demonstrated an inverse association in HC between Sniffin' Sticks test score and intake of anti-inflammatory medication and medication for high cholesterol levels. In the spice odor identification test, a trend towards an inverse association between medication intake for high cholesterol levels and hypertension and test score was observed in HC. Sensory (both olfactory and gustatory) deficits have indeed been reported for these medication types [26]. The negative effects of certain medication types (e.g., anti-inflammatory medication of medication for high cholesterol levels) may be the result of the medications interacting with the chemosensory signal transduction pathways. Furthermore, the change in chemosensory functioning due to medications can be exacerbated by taste and smell deficits associated with aging [26]. As our study population consisted of people over the age of 55 years, this may explain these results. Studies have demonstrated that olfactory dysfunction is associated with an increased risk of depression [27], potentially explaining the observed trend towards an inverse association between antidepressant intake and Sniffin' stick score in total participants in our results. Furthermore, other mental disorders, such as apathy, that frequently occur in PD have been reported to predict olfactory dysfunction in PD [28]. However, only 10.8% of our participants took antidepressants, and therefore, the interpretation of this association should be done with caution.

Consistent with previous findings [25, 29, 30], both male sex and increasing age were related to reduced olfaction in both tests. Although statistically significant, the correlation between age and different olfactory identification test scores in all participants was weak to moderate, with correlation coefficients ranging between −0.37 and −0.18.

Our results also showed a trend towards an association between self-estimated sense of smell and spice odor identification test score in HC, but not in PD. Similarly, Leonhardt et al. showed that PD patients, even though aware of their hyposmia, overestimated their sense of smell [31].

Similar to other studies, our results demonstrated a difference in recognition among the Sniffin' Sticks odorants by PD patients [15, 25, 32–34]. Our results demonstrated orange, fish, and peppermint to be the three odorants best identified by PD patients. In German PD patients, the three best identified odorants were garlic, orange, and rose [25]; in Dutch PD patients, they were fish, garlic, and clove [32]. Brazilian PD patients best identified fish, garlic, and banana odorants [33], whereas Chinese PD patients recognized garlic, orange, and peppermint the best [34]. There is some overlap in well-recognized odorants, which is consistent with previously reported results [15]. For instance, Millar Vernetti et al. reported a set of olfactory stimuli, consisting of banana, clove, coffee, fish, garlic, mint, and orange, with similar responses across different countries in PD patients and healthy volunteers [15]. However, it seems the top three odorants by PD patients recognized that Sniffin' Sticks odorants vary between countries. These differences probably can be attributed to cultural and/or environmental factors. It has been reported that cultural differences may account for the variation in olfactory identification test results across different countries. This may be related to differences in odorant exposure, for example, because of local cuisine and eating habits [15]. Furthermore, our results demonstrated a similar pattern of odor identification in PD patients and HC, further confirming the role of cultural factors in odor identification, as PD patients and HC had a similar cultural background (they were from the same region in Belgium (Flanders) and were likely to be exposed to similar types of foods).

Our results confirm the findings reported by Hähner et al. 2013, and their results demonstrated that the observed selective hyposmia in PD was not pathology-specific. They observed the same pattern of olfactory identification in PD patients and patients with non-PD hyposmia (with apple, cinnamon, coconut, honey, and pizza being the least recognized odors in both groups), similarly to what we observed a similar pattern between PD patients and HC [14]. The results by Mahlknecht et al. from 1,351 participants (including 646 PD patients) also provided evidence against the presence of pathology-specific selective hyposmia in PD [24].

Both HC and PD patients had more difficulty in recognizing the *real-life* spice odorants of the spice odor identification test, compared with the Sniffin' Sticks. This indicates that Sniffin' Sticks olfactory stimuli are more easily identified, potentially because of the higher odorant concentration. Unfortunately, we were not able to obtain information on the composition or concentration of the olfactory stimuli used in the Sniffin' Sticks, limiting us to investigate this difference further. Although we cannot confirm a higher odorant concentration in the Sniffin' Sticks, studies have demonstrated higher odor detection thresholds in PD patients compared with HC and in aged volunteers compared with younger volunteers [35, 36]. Due to more response options in the spice odor identification test, the test is considered more difficult and leads to guessing the odorant (chance success rate is 1/17 (6%) versus 1/4 (25%) for the Sniffin' Sticks) [20].

Our results demonstrated that garlic was better recognized than the other spices by both PD patients and HC. Similarly, in the Sniffin' Sticks test garlic was relatively well recognized by both population groups. The characteristic garlic aroma is attributed to the presence of sulfur compounds [37]. Humans detect the smell of volatile sulfur compounds very sensitively [38, 39], which may explain the relative high identification of garlic in both odor identification tests. In the spice odor identification test, cinnamon and clove were well recognized by HC compared with other spices. Remarkably, clove was also well recognized by HC in the Sniffin' Sticks test, whereas cinnamon was one of the least recognized Sniffin' Sticks odorants by HC. Again, this indicates a difference in the odor identification of chemical olfactory stimuli compared with real-life spice odorants.

In contrast to Knaapila et al., mean pleasantness and familiarity of the spice odorants were not correlated [19]. This might be explained by the fact that there was no large intervariability in mean pleasantness and mean familiarity scores between the odorants. However, we saw an overall association between pleasantness and familiarity scores, regardless of odorant. Although no correlation was observed between mean familiarity of an odorant and its mean identification, our results demonstrated an overall association between familiarity and identification (regardless of odorant), confirming previous findings [19, 40]. This is another explanation for the high identification of garlic, as it was the odorant with the highest familiarity reported by both PD patients and HC. Also, in Chinese and Brazilian participants, garlic was considered as a familiar scent, demonstrating its global use [33, 41].

Mean pleasantness of an odorant was not correlated with its mean identification, in contrast to the results of Knaapila et al. but consistent with findings from Appleton and Smith [19, 42]. Again, this might be explained by the low intervariability in mean pleasantness scores of odorants and the high intervariability in mean identification of odorants. However, an overall association between pleasantness and identification, regardless of odorant, was found. The pleasantness of flavor is of course important in food intake [43, 44]. Olfactory impairment may impact PD patients' food intake and body weight with an increased risk of malnutrition. In fact, PD patients actually have a higher risk of malnutrition [8, 45]. However, the underlying mechanism of malnutrition in PD is not clear. It has been hypothesized that the presence of olfactory impairment may identify PD patients at risk of malnutrition [46]. However, other studies suggest that weight loss and malnutrition are a consequence of disease progression, rather than reduced food intake [8, 47, 48]. Still, the olfactory impairment may decrease both food palatability and food enjoyment in PD patients. Tackling olfactory impairment and increasing food palatability could be realized by a better flavor identification, and the latter is associated with increased flavor pleasantness in aged participants [42]. Thus, an aroma or flavor booster should be familiar, thereby increasing potential identification and pleasantness to the patient, to potentially impact food enjoyment and food intake. Different studies have demonstrated that flavor enhancement increases food intake and food liking in aged participants [49–51].

The use of aroma or flavor boosters has not yet been evaluated in PD patients but may increase food enjoyment and food intake. A feasible strategy could be to use herbs and spices, or their purified aroma extracts as flavor enhancers, based on the patient's preference and familiarity through frequent previous use. If such an intervention showed positive results in PD patients, a patient-tailored approach may be useful to improve QoL by diet optimization.

This study has some limitations. The sample size is relatively small, specifically for the spice odor identification test due to participants' reluctance because of the difficulty and the longer duration of the test, thereby restricting external validity. Smoking was not an exclusion criterion, while it alters olfactory function. However, the proportion of participants that smoked was low (approximately 3% of all participants), limiting its potential confounding effects. HC was not matched for sex with PD patients, creating a disbalance in the ratio of men to women between HC and PD patients. Although our results regarding the effect of sex and PD on olfaction are in line with previous studies, they could be potential confounding variables, which may have impacted our results. This study still provides useful information regarding food odor recognition in PD patients, with potential implications for the use of aroma or flavor boosters in this population. The associations between participants' characteristics, including age and medication intake, and both olfactory identification test scores are based on explorative univariate analyses. The Hoehn and Yahr scale could only be collected in 22 PD patients, because of the way of recruitment (through patient organizations). The association between the Hoehn and Yahr scale and the olfactory identification test scores was therefore based on a limited sample size. These associations and the explorative analyses should be interpreted with caution and further investigated in a larger sample size.

## 5. Conclusion

This study demonstrates that some food odorants, both Sniffin' Sticks as natural odorants, are still recognized by PD patients. Sniffin' Sticks odorants were better recognized compared with *real-life* odorants, by both HC and PD patients. However, both the Sniffin' Sticks as the real-life odorant of garlic were well recognized by 47.5% and 38.2% of PD patients, respectively, probably due to their familiarity. This makes garlic an interesting candidate aroma or flavor booster for PD patients, although it scored only averagely on odorant pleasantness. Of course, perceived odorant pleasantness varies between individuals, indicating a need for a patient-tailored approach for aroma/flavor booster development. The effects of flavor enhancement in PD, however, regarding its effects on food enjoyment and food intake, need research.

## Figures and Tables

**Table 1:** Participants' characteristics.

	Part I			Part II^a^		
	PD (*n* = 80)	HC (*n* = 105)	*p* value	PD (*n* = 34)	HC (*n* = 53)	*p* value
Men/women	49/31	36/69	<0.001	22/12	20/33	0.03
Age (years), mean ± SD	68.4 ± 7.7	68.1 ± 8.0	0.67	68.7 ± 8.5	67.0 ± 8.5	0.38
Duration of disease (years), mean ± SD	8.1 ± 7.0	—	—	5.9 ± 6.1	—	—
Duration of disease (years), minimum-maximum	1–30	—	—	1–30	—	—
Hoehn and Yahr scale^b^, mean ± SD	2.2 ± 1.1	—	—	2.2 ± 1.1	—	—
Hoehn and Yahr scale^b^, minimum-maximum	1–5	—	—	1–5	—	—
BMI (kg/m^2^), mean ± SD	26.5 ± 4.5	27.9 ± 4.9	0.11	27.3 ± 5.3	28.3 ± 4.4	0.38
MNA-SF score, mean ± SD	—	—	—	12.2 ± 2.0	13.3 ± 1.2	0.003
Normal (%)	—	—	—	70.6	96.0	
At risk of malnutrition (%)	—	—	—	26.5	4.0	
Malnourished (%)	—	—	—	2.9	0.0	
Smoker/non-smoker	1/78	5/100	0.37	0/34	0/53	1.00
Sniffin' Sticks test score, mean ± SD	6.2 ± 3.1	10.3 ± 2.5	<0.001	6.5 ± 3.0	10.4 ± 2.5	<0.001
Anosmia (%)	73.8	21.9	—	73.5	22.6	—
Hyposmia (%)	23.8	43.8	—	23.5	39.6	—
Normosmia (%)	2.5	34.3	—	2.9	37.7	—
Spice odor identification test score, mean ± SD	—	—	—	1.7 ± 1.3	4.5 ± 2.3	<0.001
Self-estimated sense of smell	—	—	—			<0.001
Very good (%)	—	—	—	11.8	15.1	
Good (%)	—	—	—	11.8	39.6	
Mediocre (%)	—	—	—	32.4	35.8	
Bad (%)	—	—	—	35.3	9.4	
Very bad (%)	—	—	—	8.8	0.0	
Medication						
Parkinson's medication (%)	91.1	—	—	97.1	—	—
Antidepressants (%)	15.2	7.6	0.16	20.6	7.5	0.1
Medication for high blood pressure (%)	26.6	36.2	0.22	35.3	45.3	0.48
Medication for high cholesterol levels (%)	13.9	23.8	0.14	17.6	39.6	0.05
Anti-inflammatory drugs (%)	7.6	12.4	0.42	11.8	15.1	0.76

^a^participants of part II also completed part I; SD, standard deviation. ^b^Hoehn and Yahr scale data were collected in 22 PD patients, and these PD patients completed both part I and part II of the study.

## Data Availability

The datasets generated during and/or analyzed during this study are available in the Figshare repository, 10.6084/m9.figshare.16415667.
